# Synthesis and Tribological Studies of Branched Alcohol Derived Epoxidized Biodiesel

**DOI:** 10.3390/ma8105326

**Published:** 2015-09-24

**Authors:** Qinggong Ren, Jingjing Pan, Jie Zhou, Yinna Na, Changle Chen, Weimin Li

**Affiliations:** 1 School of Pertrochemical Engineering, Changzhou University, Changzhou 213164, China; qgren@cczu.edu.cn (Q.R.); pjjchangzhou@gmail.com (J.P.); jiezhouchang@gmail.com (J.Z.); 2 Chinese Academy of Science Key Laboratory of Soft Matter Chemistry, Department of Polymer Science and Engineering, University of Science and Technology of China, Hefei 230026, China; nayinna.ustc@gmail.com

**Keywords:** epoxidized biodiesel, ring-opening reaction, kinetics, lubrication

## Abstract

The optimization and kinetics of the ring-opening reaction of an epoxidized biodiesel (epoxidized rapeseed oil methyl ester) (EBD) with 2-ethyl hexanol (2-EH) were studied. The determined optimum conditions were 4:1 2-EH/oil molar ratio, 90 °C, 18 h, and 7 wt % of Amberlyst D001 (dry) catalyst; the product’s oxirane oxygen content was 0.081% with 38.32 mm^2^/s viscosity at 40 °C. The catalyst retained its high catalytic power after recycling five times. Furthermore, the determined non-catalyzed activation energy was 76 kJ·mol^−1^ and 54 kJ·mol^−1^ with the D001 resin catalyst. The product’s chemical structure was investigated through FT-IR and ^1^H NMR. The viscosity, flash point, pour point, and anti-wear properties of the product were improved compared with those of epoxidized biodiesel.

## 1. Introduction

Renewable resources are increasingly important for many fields including the development of environmentally friendly lubricants. Mineral oils are widely used in various lubricants. However, residual mineral oils are retained for a very long time in water or soil because of their poor biodegradability, creating serious environmental issues [1,2]. Furthermore, synthetic esters are used with great biodegradability. Unfortunately, their high cost prevents their general application. Therefore, development of alternative environmentally friendly lubricants is highly desired [3,4,5]. Vegetable oil has many advantages, such as renewability, non-toxicity, high viscosity, high biodegradability, low volatility, and low price, making it a promising alternative [6]. However, vegetable oil contains a large number of C=C double bonds, which produce unfavorable properties, such as high acid content and poor oxidative stability, thus greatly limiting its application as a lubricant.

Many strategies are applied to reduce unsaturation in vegetable oil, including selective hydrogenation [7,8,9], epoxidation [10,11,12], and esterification [13]. In selective hydrogenation, hydrogen is added to the C=C double bond to improve oxidative stability. However, harsh reaction conditions and special equipment are required [14], making large-scale industrial production difficult. Esterification is a complex and lengthy process with low yield. Epoxidation involves the oxidization of the C=C double bonds with peroxy acid under mild conditions in a simple reaction process. Unfortunately, epoxidation leads to melting temperature increase, limiting its practical application. Epoxidized biodiesel is a good chemical intermediate containing a reactive oxirane moiety, which can be easily transferred to other functional groups.

The ring-opening reaction of epoxidized vegetable oil with various alcohols forms polyol derivatives [15,16,17], with a potential to decrease the melting temperature and improve the oxidative stability [18,19,20,21]. For example, Madankar *et al*. showed that the pour point and tribological property of rapeseed oil’s ring-opening product were improved compared with those of epoxidized vegetable oil [22]. Guo *et al*. performed a kinetic study of the epoxidized palm oil reaction with acetic acid [23], and Lin *et al*. studied the reaction of epoxidized soybean oil with methanol [24], whose activation energy was 78.56 (±1.63) kJ·mol^−1^. However, the ring-opening reaction of EBD with 2-EH received very limited attention in the past. Bantchev *et al*. reported Amberlyst 15-catalyzed reactions of epoxidized alkyl soyate with different alcohols [25]. Very recently, Angelici *et al*. reported similar reactions with Amberlyst 15 catalysts [26]. However, the reaction kinetics was not investigated in both cases. Additionally, the tribological properties of the ring-opening products were studied in detail. The 2-EH branching structure might create interesting properties in the product, such as a lower melting temperature and better lubricating characteristics. In this study, we demonstrate that Amberlyst D001 (dry) can efficiently catalyze the ring-opening reaction of EBD with 2-EH to generate 2-EHBD (the product from the ring-opening reaction of epoxidized biodiesel with 2-ethyl hexanol). Amberlyst D001 is a high cross-linked, macroporous strong-acid cation-exchange resin based on polystyrene sulfonate and efficiently catalyzes esterification reactions [27]. Moreover, the reaction kinetic model is derived on the basis of this detailed kinetics study. Furthermore, we provide first-time evidence that the presence of a catalyst reduces the activation energy in this type of reaction. Finally, tribological studies show that the ring-opening product possesses good viscosity, high flash point, low pour point, and a small steel ball surface wear-scar diameter.

## 2. Results and Discussion

### 2.1. Reaction Conditions

The influence of the reaction temperature, reaction time, amount of catalyst (wt %), and 2-EH/oil molar ratio on the oxirane oxygen content and the 40 °C viscosity of the product is presented in the following sections.

#### 2.1.1. 2-EH/oil Molar Ratio

**Figure 1:**
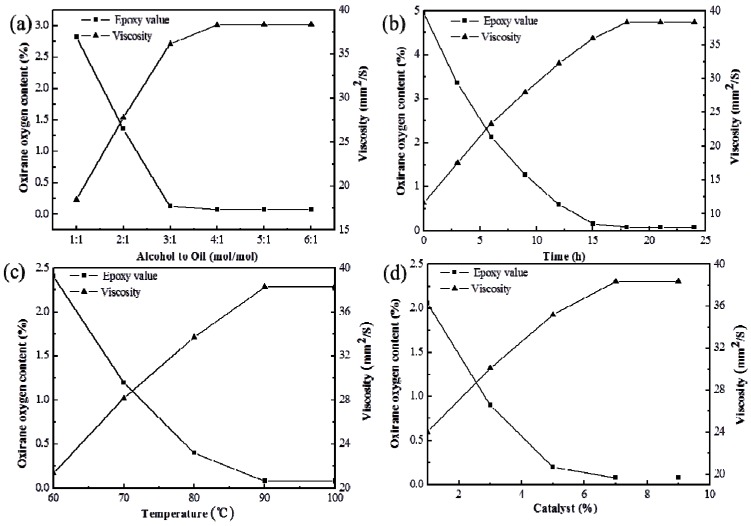
(**a**) Effect of the alcohol/oil molar ratio on the oxirane oxygen content and viscosity of the product. Experimental conditions: 7 wt % Amberlyst D001 (dry), 90 °C, 18 h; (**b**) Effect of the reaction time on the oxirane oxygen content and the viscosity of the product. Experimental conditions: 2-EH/oil ratio of 4:1, 7 wt % Amberlyst D001 (dry), 90 °C; (**c**) Effect of the reaction temperature on the oxirane oxygen content and viscosity of the product. Experimental conditions: 2-EH/oil ratio of 4:1, 7 wt % Amberlyst D001 (dry), 18 h; (**d**) Effect of the catalyst on the oxirane oxygen content and viscosity of the product. Experimental conditions: 2-EH/oil ratio of 4:1, 90 °C, 18 h.

The influence of the 2-EH/oil molar ratio on the oxirane oxygen content and viscosity at 40 °C is investigated (Figure 1a). The oxirane oxygen content decreases and the viscosity increases with increasing 2-EH/oil ratio, although they remain unchanged when the 2-EH/oil ratio increases from 4:1 to 5:1 or 6:1, suggesting the completion of the reaction. Therefore, the 4:1 2-EH/oil ratio is selected for the following studies, with an oxirane oxygen content at 0.081% and 38.31 mm^2^/s viscosity at 40 °C.

#### 2.1.2. Reaction Time Effect

At 4:1, 7 wt % 2-EH/oil ratio with Amberlyst D001 (dry), and 90 °C, we evaluate the influence of the reaction time on the oxirane oxygen content and the 40 °C viscosity (Figure 1b). The oxirane oxygen content decreases and the viscosity increases with the reaction time. After 18 h, the changes in the oxirane oxygen content and the 40 °C viscosity are very small, suggesting the completion of the reaction. Hence, 18 h is chosen as the optimal reaction time, producing 0.081% oxirane oxygen content and 38.32 mm^2^/s viscosity at 40 °C.

#### 2.1.3. Reaction Temperature Effect

We investigate the effect of the reaction temperature on the oxirane oxygen content and the 40 °C viscosity at a 4:1 2-EH/oil ratio, 18 h reaction time, and catalysis through 7 wt % Amberlyst D001 (dry) (Figure 1c). The oxirane oxygen content decreases and the viscosity increases with increasing reaction temperature. Above 90 °C, however, the changes in the oxirane oxygen content and the 40 °C viscosity become negligible, indicating the completion of the reaction. Therefore, 90 °C was selected as the optimal reaction temperature, with a 0.081% oxirane oxygen content and a 38.32 mm^2^/s viscosity at 40 °C.

#### 2.1.4. Catalyst Amount Effect

The influence of the catalyst amount on the oxirane oxygen content and the viscosity at 40 °C was investigated at a 4:1 2-EH/oil ratio, 90 °C reaction temperature, and 18 h reaction time (Figure 1d). An increase in the catalyst amount led to a faster reaction and a lower oxirane oxygen content. After a certain amount of Amberlyst D001 (dry), the oxirane oxygen content and the viscosity of the product remained constant. Therefore, 7 wt % was chosen as the optimal catalyst amount, producing an oxirane oxygen content of 0.082% and a viscosity at 40 °C of 38.34 mm^2^/s.

### 2.2. Catalyst Recycling

After the reaction, the Amberlyst D001 (dry) catalyst was filtered, washed with 100 mL water and 100 mL alcohol, and refluxed with alcohol for 4 h to remove any impurities from the catalyst pores. Then, the catalyst was dried in a vacuum oven at 60 °C for 4 h and reused. The catalyst was tested five times under the same reaction conditions. The oxirane oxygen content of the starting material is *ca*. 5%; a low content implies greater conversion. The results are observed in Table 1. Clearly, Amberlyst D001 (dry) exhibits good chemical stability, which is retained after five cycles.

**Table 1:** Amberlyst D001 (dry) reusability. Experimental conditions: 2-EH/oil ratio of 4:1, 7 wt % Amberlyst D001 (dry), 90 °C, 18 h.

Reusability	One	Two	Three	Four	Five
Oxirane oxygen content (%)	0.081	0.126	0.182	0.243	0.264

### 2.3. Oxirane Cleavage Reaction Kinetics

To investigate the intrinsic dynamics of the ring-opening reaction with 2-EH and explore the reaction mechanism, a kinetic study was conducted. A kinetic model of the ring-opening reaction was established, and the chemical reaction kinetic equation as well as the activation energy was determined.

#### 2.3.1. Kinetic Model

The epoxy group of the epoxidized biodiesel is a ternary ring, which reacts easily with alcohol and acid. Generally, the kinetic model of EBD with 2-EH can be expressed as follows:

(1) $$r=-\frac{d\left[Ep\right]}{dt}=k{\left[Ep\right]}^{m}{\left[2-EH\right]}^{n}$$

where *r* is the rate of the reaction, mol·L^−1^·h^−1^; [Ep] is the molar concentration of the epoxy group, mol·L^−1^; [2-EH] is the 2-EH molar concentration, mol·L^−1^; *m* and *n* are the reaction orders with respect to the epoxy group and 2-EH, and *k* is the rate constant of the ring-opened reaction.

#### 2.3.2. Reaction Rate Constant

If the molar concentration of 2-EH in a set of experiments is in large excess as compared to that of the oxirane oxygen, the rate equations can be simplified to:

*r* = *k′*[Ep]*^m^* (2)

where *k*′ is the rate constant when 2-EH is in large excess, *i.e.*,

*k′* = *k*[2-EH]*^n^* (3)

**Figure 2:**
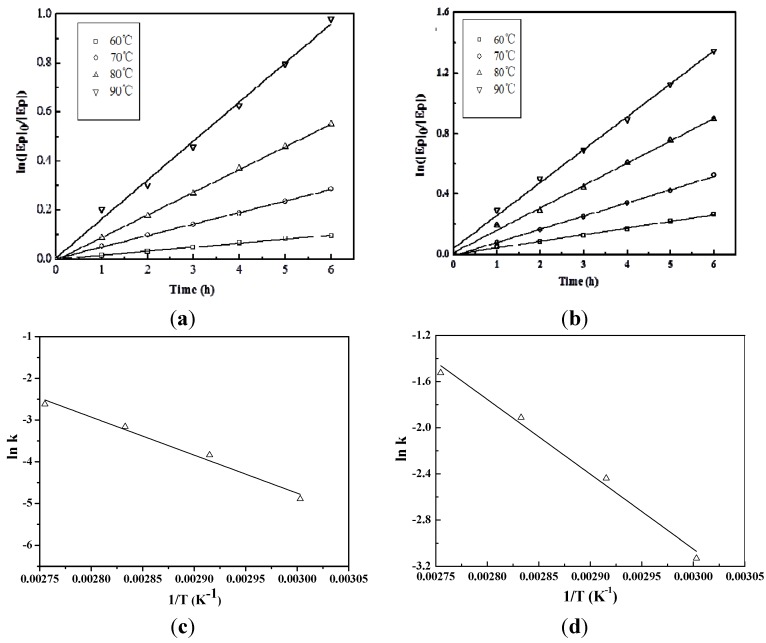
(**a**) Determining the rate constant *k′* at different temperatures without catalyst. Experimental conditions: 2-EH/oil ratio 10:1, 6 h reaction time, without catalyst; (**b**) Determining the rate constant *k′* at different temperatures with the D001 resin catalyst. Experimental conditions: 2-EH/oil ratio 4:1, 6 h, 7 wt % Amberlyst D001 (dry); (**c**) Determining the activation energy (*Ea*) without a catalyst; (**d**) Determining the activation energy (*E*_a_) with the D001 resin catalyst.

EBD (100 g, 0.3094 mol epoxy group) was used at 60 °C, 70 °C, 80 °C, and 90 °C with a three-necked round-bottom flask in the presence of the D001 resin catalyst and without the catalyst. The aliquots of the reaction mixture were obtained at 1-h intervals during the reaction. The samples were immediately analyzed by GC (Figure 2a). A linear relationship is observed between ln([Ep]_0_/[Ep]) and the reaction time, indicating first-order kinetics for the oxirane concentration. First order kinetics is also observed for similar reactions with vegetable oils [24,28]. Without the catalyst, the rate constant *k′* (*i.e.*, the linear regression slope) is 0.0164 h^−1^, 0.0469 h^−1^, 0.0925 h^−1^, and 0.1591 h^−1^. Applying the D001 resin catalyst, the rate constant *k′* (*i.e.*, the linear regression slope) is 0.0437 h^−1^, 0.0873 h^−1^, 0.1478 h^−1^, and 0.2178 h^−1^.

#### 2.3.3. Activation Energy *E_a_*

The activation energy *E_a_*, as an important parameter in the reaction kinetic equation, is determined by the reaction rate constant and the temperature. In general, their relationship can be expressed by the Arrhenius formula:

Integral index formula: *k* = *A*e^−*Ea*/RT^ (4)

Integral logarithmic formula: ln*k* = ln*A* − *E_a_*/RT (5)

A linear relationship is observed between ln*k* and 1/*T* (Figure 2c) with a slope (−*E_a_*/R) of −9.116 × 10^3^. Therefore, the activation energy (*E_a_*) is 76 (±7) kJ·mol^−1^. In the catalyzed reaction, the slope (−*E_a_*/R) is −6.479 × 10^3^ with an *E_a_* of 54 (±4) kJ·mol^−1^ (Figure 2d).

#### 2.3.4. Reaction Kinetics

Lin *et al*. determined the reaction rate equation in the oxirane cleavage of epoxidized soybean oil with methanol without a catalyst (*r* = *k*[Ep][MeOH]^2^), and the activation energy was 78.56 (±1.63) kJ·mol^−1^ [24]. However, they did not study the catalyzed reaction, which is the case in most applications. In this work, we clearly demonstrate that the activation energy with a catalyst is much lower than that without one (Table 2).

**Table 2:** *E_a_* and rate equation of different ring-opening reactions.

Oil	2-EHBD (D001 resin)	2-EHBD (without a catalyst)	ESO-Me [24] (without a catalyst)
*E*a (kJ·mol^−1^)	54 (±4)	76 (±7)	78.56 (±1.63)

### 2.4. Product Characterization

**Figure 3:**
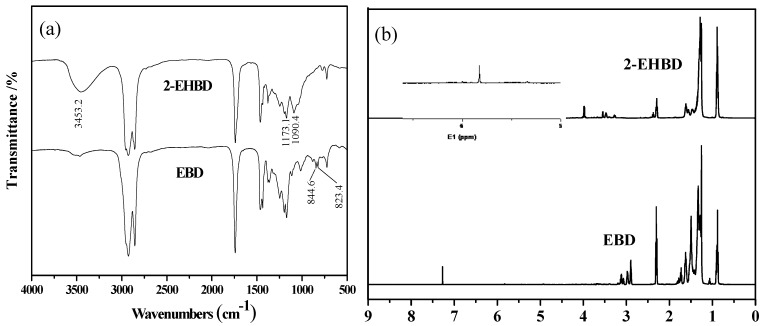
(**a**) FTIR spectra of the epoxidized biodiesel and the ring-opening product; (**b**) ^1^H NMR spectral of the epoxidized biodiesel and the ring-opening product.

The FTIR spectra of the product and the raw material are presented in Figure 3a. There are some common peaks in the FTIR spectra, at 2926 cm^−1^ to 2855 cm^−1^ (methylene asymmetric stretching), 1741 cm^−1^ (triglycerides carbonyl stretching), 1464 cm^−1^ (CH_2_ bending vibration), 1377 cm^−1^ (CH_3_ symmetrical bending vibration), and 724 cm^−1^ (CH_2_ rocking vibrations). The epoxide peaks between 823.4 cm^−1^ and 844.6 cm^−1^ do not appear in the product. Additionally, the broad and strong absorption peak of the alcoholic hydroxyl at 3453 cm^−1^ confirms the presence of the ring-opening product. Moreover, C-O absorption peaks of aliphatic alcohols and aliphatic ethers can be found between 1200 cm^−1^ and 1025 cm^−1^.

The ^1^H NMR spectra of EBD and 2-EHBD are presented in Figure 3b. There are some common ^1^H NMR peaks at δ 2.25–2.5 ppm for CH_2_ protons α to >C=O, 1.68–1.85 ppm for CH_2_ protons connected with the epoxy groups, 1.15–1.28 ppm for all other CH_2_ protons, 0.8–1.0 ppm for terminal CH_3_ protons, and 2.8–3.2 ppm for CH protons of the epoxy group. The epoxy peaks at 2.8–3.2 ppm decrease, and a new CH_2_ proton peak on the side chain (–CH–O–CH_2_–CH(C_2_H_5_)(CH_2_)_3_CH_3_) appears at 3.95–4.01 ppm. The presence of a peak at δ 5.81 suggests a furan product in our system, which was previously reported by Bantchev *et al*. [25]. However, we could not determine the presence or absence of a tetrahydrofuran product, which was previously reported by Angelici *et al*. [26].

### 2.5. Comparison of the Physical and Chemical Properties

The physical and chemical properties of the epoxidized biodiesel and the ring-opening product are compared in terms of viscosity, flash point, viscosity index, pour point, wear scar diameter, and the *P*_B_ (maximum non-seizure load) (Table 3). The product’s viscosity at 40 °C is considerably greater than that of EBD. The trend is maintained at 100 °C. Probably, the ring-opening reaction introduces polar groups, such as hydroxyl groups, increasing the oil’s viscosity. The viscosity index is smaller after the ring-opening reaction, suggesting better performance at low temperatures. The product’s flash point is greater than that of EBD, enabling its application as lubricating oil. In addition, the oxidation stability is significantly improved after the reaction. In the anti-wear experiment, the wear scar diameter and the *P*_B_ value of ring-opening product modifying the lubricant base oil is superior to that of the epoxidized biodiesel. The lubricant with the ring-opening product can efficiently form a thicker protective film on the metal surface and improve the friction performance.

**Table 3:** Pour point, kinematic viscosity, flash point, oxidative stability, and tribological properties of the epoxidized biodiesel and the ring-opening product.

Sample	Epoxidized biodiesel	Ring-opening product
Pour point/°C	0	−10
Viscosity of 40 °C/(mm^2^·s^−1^)	8.9	38.30
Viscosity of 100 °C/(mm^2^·s^−1^)	3.5	6.6
Viscosity Index	253	124
Flash point/°C	185	230
Oxidation induction time/min	16	22
WS*D*^392^/mm	0.52	0.44
*P_B_*/*N*	548.8	695.8

**Figure 4:**
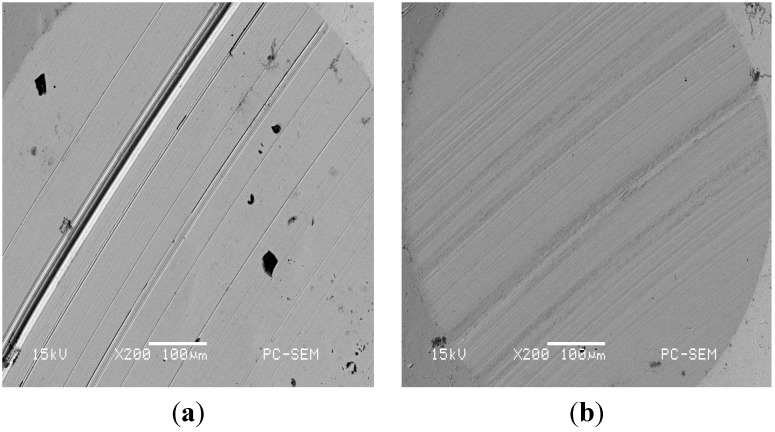
SEM image of the lubricant base stock with (**a**) EBD; (**b**) 2-EHBD.

SEM images of the stainless steel plate after the anti-wear experiment appear in Figure 4. Clearly, the lubricant base with the ring-opening product performs better that the lubricant base with EBD. The hydroxyl groups in the product can increase the oil’s thickness on the metal surface, decrease the steel ball grinding crack width, reduce the surface grinding crack, produce only a small furrow phenomenon, and improve the tribological properties of the lubricant.

## 3. Experimental Section

### 3.1. Materials

The EBD was obtained from the laboratory (Changzhou, China). Oxirane oxygen 4.95% was used, equivalent to 0.3094 mol oxirane in 100 g of EBD. Amberlyst D001 (dry) (ionic from H+) was supplied by Xin Rui Technology Co. Ltd (Wuhan, China; Size: >95%, 0.32–1.25 mm; Temperature stability: >100 °C; Weight capacity: 4.35 mEq/g). The EBD was prepared through the epoxidation procedure for canola oil [22] using hydrogen peroxide as oxidant; the oxirane value was 5.02 g/100 g. All other chemicals were purchased from Sinopharm Chemical Reagent Co., Ltd. (Beijing, China) and used without further purification.

### 3.2. Analytical Methods

The product’s oxirane oxygen content was determined according to the AOCS method Cd 9-57. The structure of the epoxidized biodiesel and the ring-opening product were identified through Fourier-transform infrared (FTIR) and ^1^H NMR spectroscopy. The FTIR spectra were obtained using a Nicolet Protege-460 FT-IR spectrometer (Thermos Nicolet Corporation, Madison, WI, USA) with KBr crystals in thin-film after 64 scans at a resolution of 4 cm^−1^. The ^1^H NMR spectra were obtained with a Bruker (Bruker Biospin, Fällanden, Switzerland) Avance III-400 spectrometer. The weight loss of the sample was measured using a Diamond DSC Thermogravimetric Analysis (PerkinElmer, Fremont, CA, USA) in the presence of air (flow rate: 20 mL/min) and a constant heating rate of 10 °C/min. The tribological tests were performed on a MRS-10A-four-ball tribometer, (Jinan Assay Group, Jinan, China).

### 3.3. Ring-Opening Reaction

**Figure 5:**
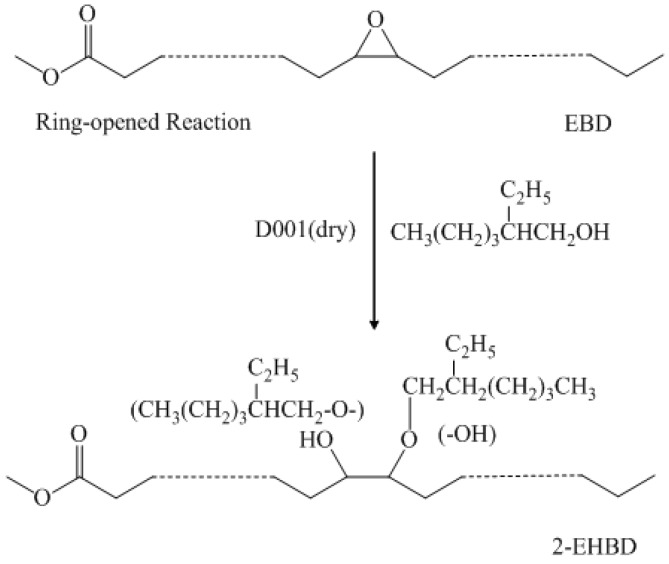
Ring-opening reaction of epoxidized biodiesel with 2-ethyl hexanol (considering there are significant amounts of methyl linoleate and methyl α-linoleate in biodiesel, the ring-opening product from bi-epoxy or tri-epoxy materials could also be present).

Epoxidized biodiesel (50.0 g), 2-EH (86.7 g, 2-EH/oil ratio of 4:1) and Amberlyst D001 (dry) (3.5 g, 7 wt % relevant to epoxidized biodiesel) were added to a 500 mL three-necked round-bottomed reaction flask (250 mL capacity), equipped with a stirrer, a thermometer, and a reflux condenser. The mixture was placed in a water bath and heated to 90 °C, while stirring for 18 h. After the reaction was completed, the mixture was poured into a separatory funnel. The sample was washed with 100 mL hot distilled water (50 °C), 50 mL hot dilute alkali (1.5%, w/w; 50 °C), and 100 mL hot distilled water (50 °C) again. Finally, the product was dehydrated over MgSO_4_, and 2-EH was removed by vacuum distillation. The reaction equation is presented in Figure 5.

## 4. Conclusions

The 2-EHBD is synthesized at high yield from the reaction of EBD with 2-EH in the presence of Amberlyst D001 (dry) catalyst. The optimum reaction conditions are 4:1 2-EH/oil molar ratio, 90 °C, 18 h reaction time, and 7 wt % catalyst. The catalyst is highly stable under the reaction conditions and can be easily recycled and reused. The kinetic model and activation energy are, furthermore, determined. The chemical structure of the product is determined by FTIR and ^1^H NMR spectrometry. The ring-opening product exhibits good viscosity, high flash point, low pour point, small steel ball surface wear-scar diameter, and excellent tribological properties.
